# The Effect of Nonessentialist Beliefs About Aging on Health Behavior Intentions

**DOI:** 10.1093/geroni/igaa057.1046

**Published:** 2020-12-16

**Authors:** Ga-Eun (Grace) Oh

**Affiliations:** Open University of Hong Kong, Kowloon, China

## Abstract

Globally, as people expect the longer life expectancy than ever before, people have increasing concerns about their health and aging. Although what people believe regarding aging can affect their health behaviors, limited research has investigated which beliefs regarding aging influence health behaviors. Previous research has shown that essentialist beliefs about aging reflect beliefs that the aging process is fixed, while nonessentialist beliefs about aging reflect the beliefs that the aging process is rather malleable. Since beliefs in nonessentialism regarding aging imply the benefits of health-promoting behaviors, we examine if manipulating nonessentialist beliefs about aging could contribute to intentions to engage in health behaviors. We also investigate if age and income might moderate the effect of nonessentialist beliefs. We conducted an experiment with a sample of American participants of varied ages (n = 599). The results showed that compared to essentialist beliefs, nonessentialist beliefs regarding aging significantly increased an intention to eat healthy food but they did not improve intentions of other health-promoting behaviors in terms of regular exercise and consumption of fruits and vegetables. Income moderated the effect of essentialist beliefs on an intention to eat fruits and vegetables. Specifically, nonessentialist beliefs had a positive effect among high-income people but rendered a negative effect among low-income people. Together, the present findings provide initial evidence that nonessentialist beliefs have a potential to promote health behaviors and call for the further investigation of the effects of educating nonessentialist beliefs on actual health behaviors and the boundary conditions of the effects.

